# Novel translucent monolithic zirconia fixed restorations in the esthetic zone

**DOI:** 10.1002/ccr3.5499

**Published:** 2022-03-06

**Authors:** Carlos Alberto Jurado, Jose Villalobos‐Tinoco, Hidehiko Watanabe, Ramon Sanchez‐Hernandez, Akimasa Tsujimoto

**Affiliations:** ^1^ Texas Tech University Health Sciences Center El Paso Woody L. Hunt School of Dental Medicine El Paso Texas USA; ^2^ Specialty Program in Periodontics National University of Rosario School of Dentistry Rosario Argentina; ^3^ Department of Restorative Dentistry Oregon Health and Sciences University School of Dentistry Portland Oregon USA; ^4^ Private Practice San Felipe Mexico; ^5^ Department of Operative Dentistry University of Iowa College of Dentistry Iowa City, Iowa USA

**Keywords:** dentistry

## Abstract

Novel translucent monolithic zirconia has improved optical properties, and it may fulfill patient's esthetic demands and overcome the chipping risk of bilayer metal‐ceramic restorations. New zirconia's microstructures allow us to mimic natural teeth.

## Graphical abstract

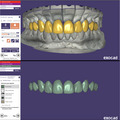

## 1 INTRODUCTION

Due to their esthetic quality and high success rate, metal‐ceramic crowns have been the restoration of choice for decades.^1^ However, all‐ceramic crowns have become more popular for esthetic cases due to their biocompatibility.^2^, ^3^ Moreover, patients' demand for esthetic restorations has also resulted in increased use of all‐ceramic restorations in anterior and posterior cases.^4^, ^5^ In a systematic review, 5 years survival rates of metal‐ceramic and all‐ceramic single crowns were reported to be quite similar, presenting 95.6% for metal‐ceramic and 93.3% for all ceramic.^6^ Among the ceramics, the use of zirconia fixed restorations has considerably increased due to the excellent mechanical properties and esthetic results.^7^, ^8^, ^9^

High strength‐oxide ceramics as the core material were introduced to improve esthetics, but concerns regarding chipping of the layering have been reported.^10^ A study showed that bi‐layered zirconia presented 24% of chipping compared to 34% of chipping to porcelain fused to metal after 3 years.^11^ Chipping and fractures of the veneering ceramic have been reported as a problem, so in recent years, the high strength monolithic zirconia with glazed and stained crowns have been evaluated for many aspects.^12^ Monolithic zirconia restorations do not have any other ceramic veneering or layers that could chip or fracture. However, this type of ceramic is monochromatic and could be too opaque, so esthetic properties are considered inferior to conventional veneered crowns. Therefore, although monolithic zirconia crowns have been widely accepted as a treatment of choice for heavy grinders and patients with parafunctional habits, their use in the esthetic zone has been minimal.

The favorable mechanical properties of zirconia have prompted extensive research.^13^ Zirconia is made of polymorphic crystals, and it is commonly categorized into three forms: tetragonal, cubic, and monoclinic. Zirconia at room temperature is monoclinic and stable. Once the temperature reaches 1170°C and 2370°C, zirconia becomes tetragonal and cubic phases, respectively.^13^, ^14^ The conventional zirconia in dentistry contains 3% of yttria to stabilize the tetragonal phase at room temperature. This 3 mol% yttria‐stabilized tetragonal zirconia polycrystal (3Y‐TZP) with tetragonal zirconia can form a transformation zone that will shield cracks.^15^ This transformation toughening contributes to a high fracture resistance to dental zirconia. Unfortunately, the major drawback of the first 3Y‐TZP is its opacity. It contained alumina to help in the sintering process in order to prevent pores. However, zirconia and alumina have different indices of refraction; therefore, alumina can decrease the light transmission.^16^ The newest version of zirconia has been made with increased yttria content. It is fabricated with 5 mol% yttria that partially stabilizes the cubic phase.^17^ This new zirconia (5Y‐ZP) in the cubic phase is more translucent than 3Y‐TZP because it is isotropic in different crystallographic directions.^18^, ^19^ This novel translucent zirconia (5Y‐ZP) has been called translucent zirconia due to its improved optical properties. The advent of the novel 5Y‐ZP promises high translucency similar to that of glass‐ceramics such as lithium disilicate; therefore, the aim of this report is to clinically evaluate esthetic results of monolithic translucent zirconia restorations in the esthetic zone.

## 2 CASE REPORT

**FIGURE 1:**
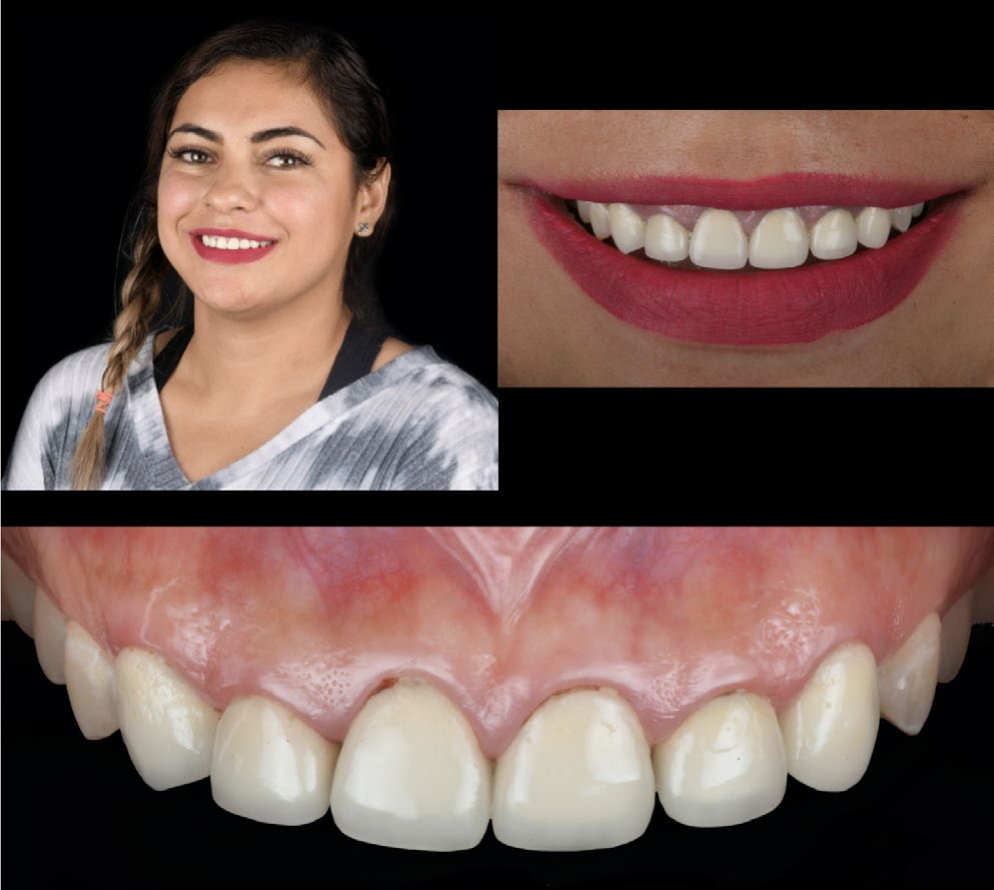
Initial clinical situation

A 35‐year‐old patient presented to the clinic with the chief complaint of disliking her anterior porcelain‐fused‐to‐metal crowns (Figure 1). The patient has been with these restorations for 6 months, and she wants to replace them. Upon the clinical evaluation, it was noticed that #6 to #11 were restored with porcelain‐fused‐to‐metal restorations. Gingival inflammation and bleeding were found around crown #8. Exposed metal collars on crowns #8, 9, and 10 were noted. Moreover, different sizes and positions of incisal embrasures caused a non‐esthetic smile line. The patient was offered to replace the porcelain‐fused‐to‐metal crowns with translucent zirconia restorations with staining and characterization provided by a dental technician. Cuspal coverage restorations were recommended for premolars due to the occlusal wear, and she agreed. Adequate diagnostic wax‐up (Geo Classic, Renfert) was performed to evaluate discrepancies between the current crowns' shape and the proposed shape of new restorations. The diagnostic wax‐up was presented to the patient, and she was pleased with it.

**FIGURE 2:**
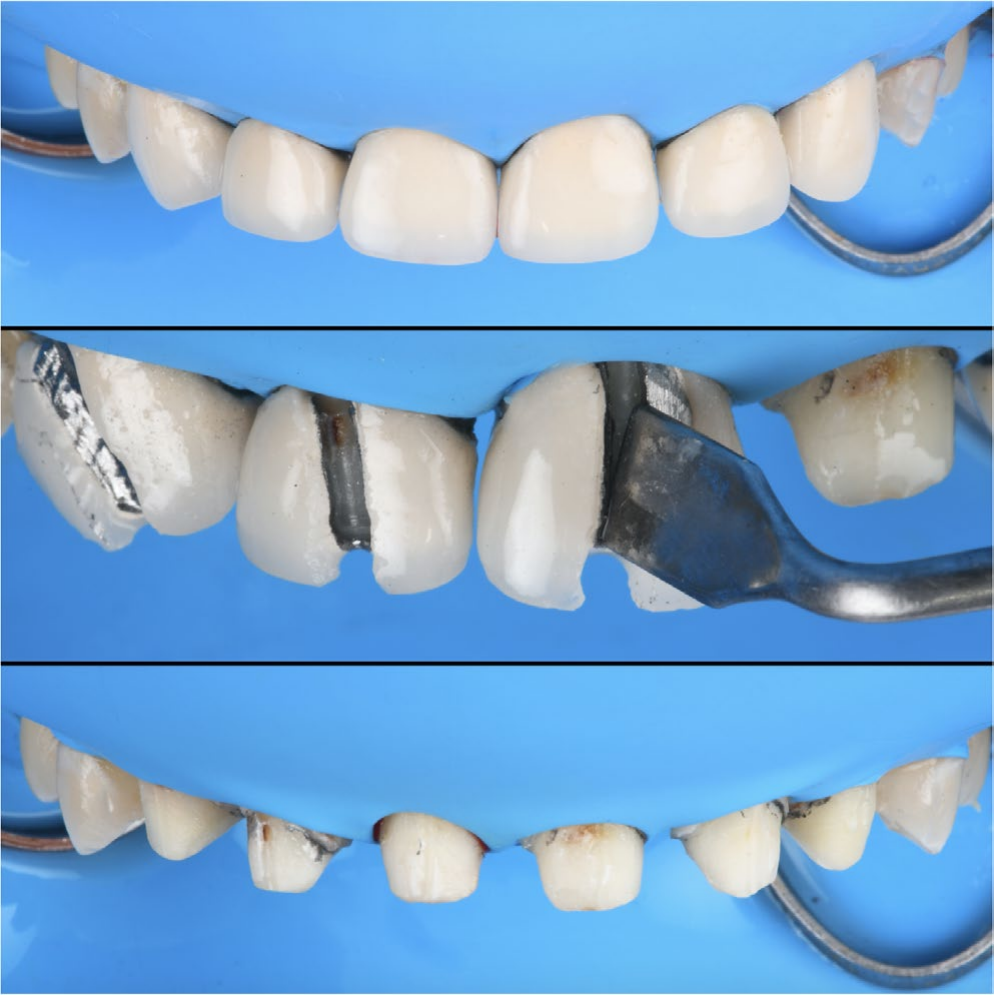
Old porcelain‐fused‐to‐metal crowns removal

**FIGURE 3:**
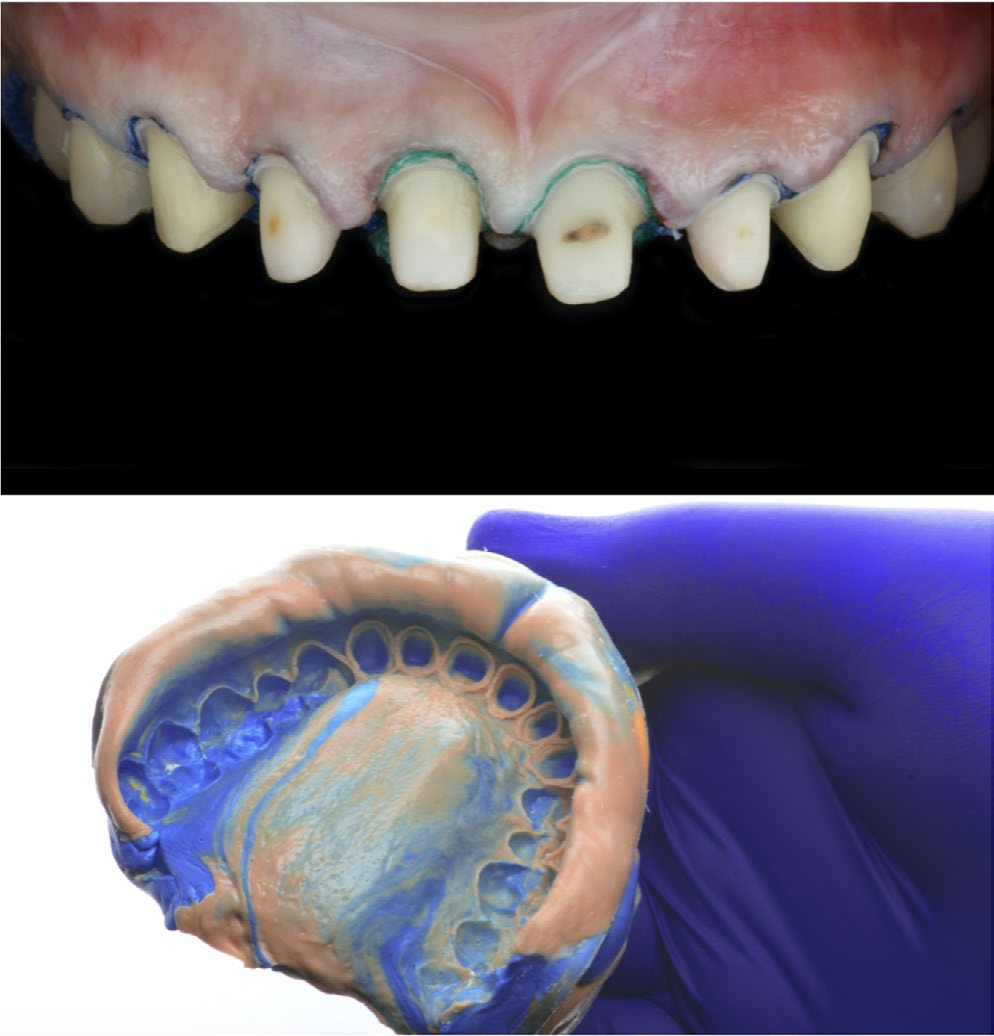
Cord packing and final impression

At the following clinical appointment, isolation was provided with a rubber dam (Dental Dam, Nic Tone, MDC Dental; Zapopan, Mexico) from #4 through #13, placing clamps on #4 and #13 (Clamps #00, Hu‐Friedy) and old porcelain‐fused‐to‐metal restorations shade A2 were removed (Figure 2), and provisional acrylic restorations were placed. At the following appointment, a double cord impression was placed, with packing a cord #2 followed by a #1 (Ultrapak, Ultradent Products Inc), and a final impression was taken using heavy‐body and light‐body consistency polyvinylsiloxane (Virtual 380, Ivoclar Vivadent) (Figure 3). Master cast and individual dies were fabricated with type IV stone (Fujirock, GC America Inc).

**FIGURE 4:**
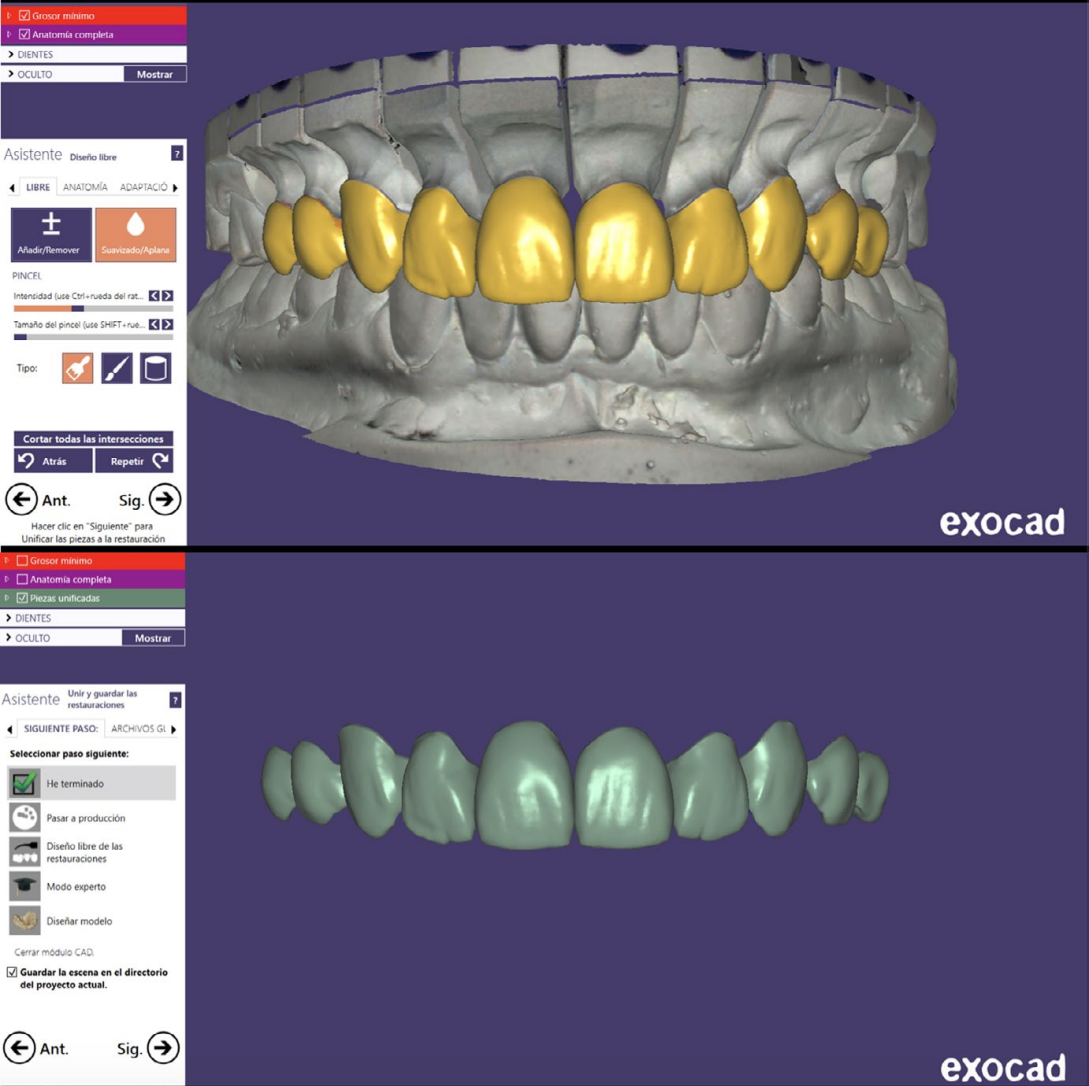
Digital design of final restorations

**FIGURE 5:**
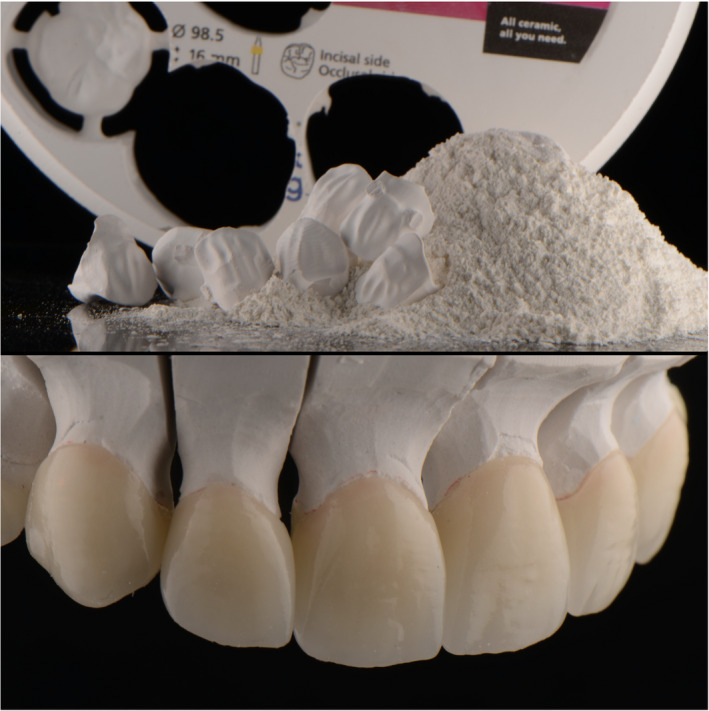
Milled and sintered restorations

**FIGURE 6:**
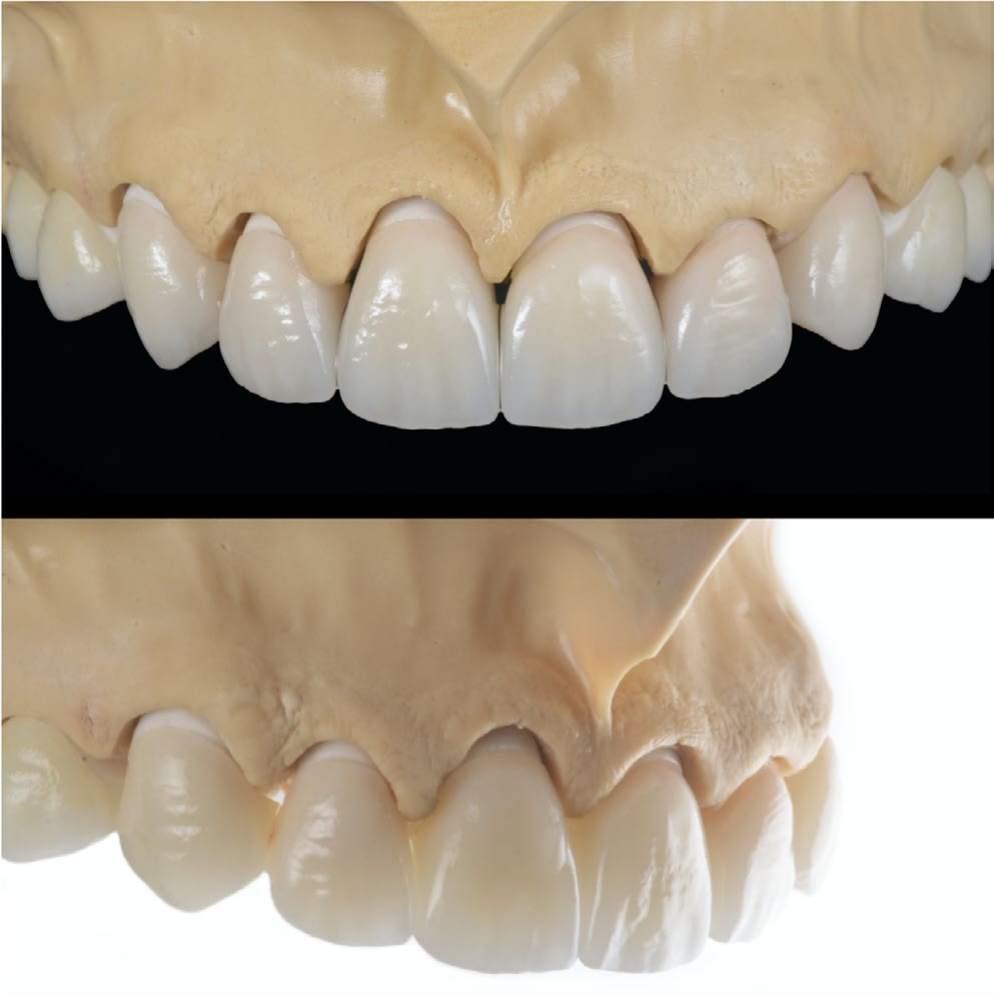
Characterization of final translucent zirconia restorations

**FIGURE 7:**
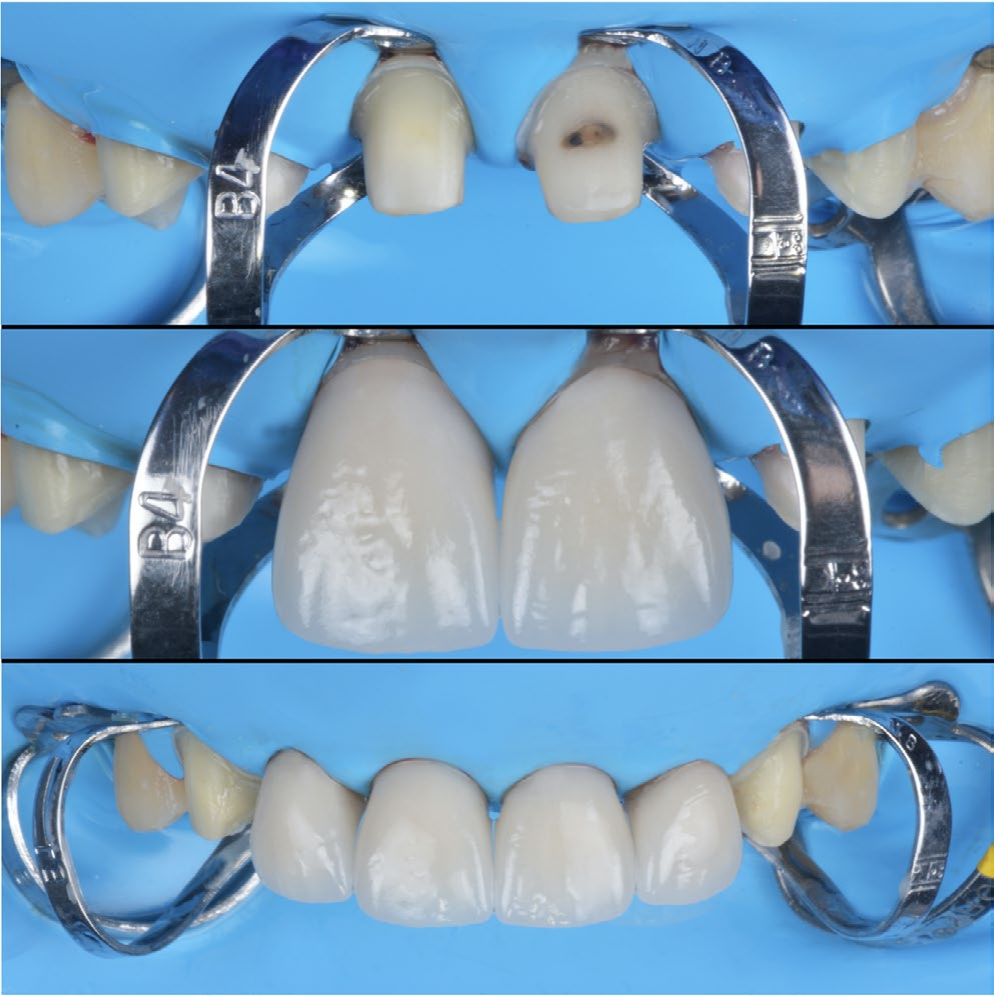
Cementation of final restorations

**FIGURE 8:**
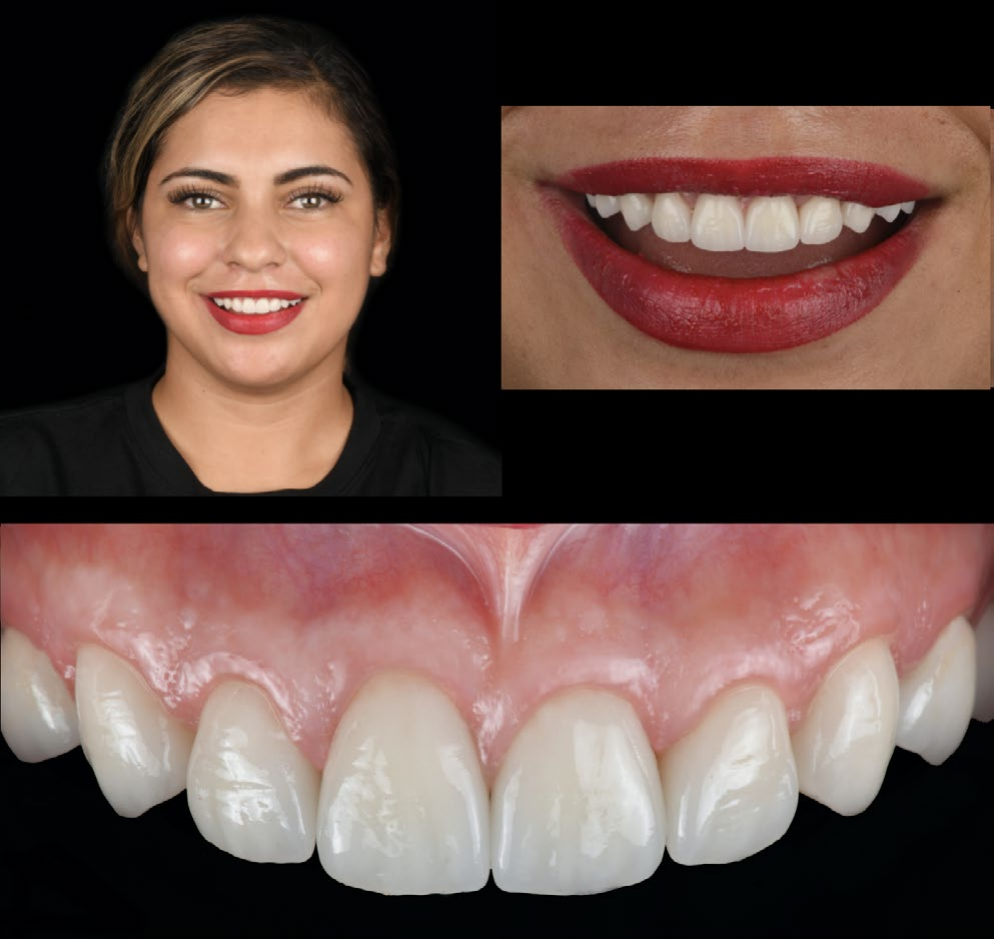
Final clinical situation

The master cast was scanned with a dental desktop 3D scanner (CS Neo, CADstar Dental Solutions GmbH), and full monolithic translucent zirconia restorations were digitally designed (Dental CAD, Exocad GmbH) following the patient's esthetic desire (Figure 4). Monolithic translucent zirconia restorations shade B1 were digitally oriented within the zirconia disc to have the incisal zone with the highest translucency, the transition zone in the middle and the less translucency area in the gingival third of the restoration, and then, they were milled out (Zenotec Select Hybrid, Wieland Dental) from zirconia (ZirCAD Prime, Ivoclar Vivadent), and restorations were glazed and sintered (CS4 Programat, Ivoclar Vivadent) following the manufacturer's recommendation (Figure 5). Restorations were stained and characterized in order to mimic natural teeth. (Figure 6). A rubber dam (Dental Dam, Nic Tone, MDC Dental; Zapopan, Mexico) was placed from the second premolar to the contralateral second premolar and retained with clamps (Clamp #00, Hu‐Friedy) for appropriate isolation. Clamps were also placed along the gingival contour of every tooth to be treated (Clamp B4, Brinker, Hygenic, Coltene), and restorations were tried‐in. Then, restorations were sandblasted with 50 microns of aluminum oxide below 2 bar pressure, followed by cleansing with a cleaning paste (Ivoclean, Ivoclar Vivadent) for 20 s. After rinsing and drying, Zirconia primer (Z‐Primer Plus, Bisco Inc) was applied to restorations' intaglio surface and air‐dried for 5 s. The teeth were treated with 29 microns of aluminum oxide and water (AquaCare Single, Velopex International), followed by 32% selective phosphoric acid etching (Uni‐Etch w/BAC, Bisco Inc) 15 s in enamel, followed by rinse for 5 s with suction. Light‐cured dental adhesive (All‐Bond Universal, Bisco Inc) was applied on the tooth surface and air‐dried to remove excess, followed by light curing for 10 s. Restorations were cemented with resin luting cement (Duo‐link Universal Shade, Bisco Inc) and light‐cured for 2 s on mesio‐facial, disto‐facial, disto‐lingual, and mesio‐lingual surfaces. Excessive cement was removed, and final light curing for 40 s was provided (Figure 7). Removing the rubber dam, we evaluated the occlusion, including excursive movements and protrusion. The patient was pleased with the contours, shape, and shade of the final translucent monolithic zirconia restorations (Figure 8). An occlusal guard was provided to the patient to wear at night to prevent damage to the restorations and existing teeth. Restorations will be monitored every 6 months during the oral hygiene appointments.

## 3 DISCUSSION

The patient presented with esthetic concerns after having porcelain‐fused‐to‐metal (PFM) restorations in the maxillary anterior region. After the initial evaluation, gingival inflammation and bleeding on the margin of crown #8 were found. The patient was informed that a conservative approach could be provided with dental prophylaxis followed by monitoring the restorations. The patient's dissatisfaction with the previous dental care outcome drove her to request the restorations' replacement without any metal and providing a harmonious smile.

Porcelain‐fused‐to‐metal crowns have been considered to be a gold standard in dentistry for a long time.^20^ However, the esthetic results of the PFM may be compromised by the metal framework because the layering porcelain is needed to mask the grayish metal shade. It may also provide esthetic problems whenever the metal collar of the PFM is exposed due to small gingival recession or simply by supragingival margin placement by clinicians. In the pretreatment clinical situation, the metal margins were seen on teeth #8, 9, and 10, and those small dark spots could be detected by the patient, resulting in esthetic concerns. We provided a new set of translucent zirconia restorations with margins located 0.5 mm subgingivally to fulfill esthetic concerns. It has been clinically accepted to have 0.25 to 0.5 mm subgingival margins to maintain healthy biologic width.^21^

Translucent monolithic zirconia restorations could be opaquer than glass‐ceramics. However, patients were looking to have a “Hollywood smile”,^22^, ^23^ requesting restorations with somewhat white and opaque so that zirconia restorations may fulfill those patient's desires. Fortunately, monolithic zirconia restorations can be characterized and stained with all anatomical features that can match a natural‐looking appearance. Moreover, the new translucent zirconia has crystals that decrease the light scattering and increase its translucency.^17^, ^18^ Recent studies have also demonstrated that novel translucent zirconia has higher fracture strength than other ceramics such as lithium disilicate^19^, ^24^ and very small chipping rate.^25^ Due to the translucent and fracture resistance zirconia's promising initial data, it was selected as a restorative material.

The workflow of these restorations was a combination of conventional and novel techniques. Conventional polyvinyl siloxane impression was taken, followed by conventional cast fabrication of dental model with type IV dental stone. Then, the cast was scanned for digital designing of restorations. Final restorations were milled out of zirconia ceramic, but a dental technician manually provided the characterization and staining. This type of restorations can be entirely fabricated with a digital workflow; however, the artistic micro details provided by a technician's hands still cannot be reproduced by the milling equipment. The software used in this case (Dental CAD, Exocad GmbH) allows for a personalized digital design of the restorations with different shapes such as square, round and ovoid and patient and clinician can see the images of the designs and select them before their fabrication. The final translucent zirconia restorations provided in this report showed appropriate esthetic and clinical performance. High esthetic results for translucent zirconia crowns in the esthetic zone can be achieved through the procedures described above, but the outcome depends on a detailed treatment plan evaluating esthetic and functional parameters while considering patients' desires. Furthermore, the patient's cooperation during treatment and post‐operative care of the restorations is crucial to achieving a positive outcome.

## 4 CONCLUSIONS

Monolithic translucent zirconia crowns in the anterior zone may provide high esthetic results similar to glass‐ceramic restorations. A dental technician must provide stain and characterization with anatomic features in the ceramic during the fabrication process.

## CONFLICTS OF INTERESTS

The authors declare that there is no conflict of interest regarding the publication of this article.

## AUTHOR CONTRIBUTION

CAJ and JVT contributed to concepts and manuscript editing. CAJ and AT contributed to design. HH and RSH contributed to definition of intellectual content. HH and JVT contributed to manuscript editing. JVT and AT contributed to literature search and manuscript review.

## ETHICAL APPROVAL

The patients described were fully informed on the method and the purpose of the case report. Written consent to participate and for publication was obtained by the patients and is available upon request.

## CONSENT

All authors have confirmed during submission that patients' consents have been signed and collected in accordance with the journal's patient consent policy.

## Data Availability

The data that support the findings of this study are available from the corresponding author upon reasonable request.
